# From Data to Causes III: Bayesian Priors for General Cross-Lagged Panel Models (GCLM)

**DOI:** 10.3389/fpsyg.2021.612251

**Published:** 2021-02-15

**Authors:** Michael J. Zyphur, Ellen L. Hamaker, Louis Tay, Manuel Voelkle, Kristopher J. Preacher, Zhen Zhang, Paul D. Allison, Dean C. Pierides, Peter Koval, Edward F. Diener

**Affiliations:** ^1^Department of Management and Marketing, The University of Melbourne, Parkville, VIC, Australia; ^2^Department of Methodology and Statistics, Utrecht University, Utrecht, Netherlands; ^3^Department of Psychological Sciences, Purdue University, West Lafayette, IN, United States; ^4^Department of Psychology, Humboldt University of Berlin, Berlin, Germany; ^5^Department of Psychology and Human Development, Humboldt University of Berlin, Berlin, Germany; ^6^Cox School of Business, Southern Methodist University, Dallas, TX, United States; ^7^W.P. Carey School of Business, Arizona State University, Tempe, AZ, United States; ^8^Department of Sociology, University of Pennsylvania, Philadelphia, PA, United States; ^9^Stirling Management School, University of Stirling, Stirling, United Kingdom; ^10^Melbourne School of Psychological Sciences, The University of Melbourne, Parkville, VIC, Australia; ^11^Department of Psychology, The University of Utah, Salt Lake City, UT, United States; ^12^Department of Psychology, University of Virginia, Charlottesville, VA, United States

**Keywords:** panel data model, Granger causality (VAR), Bayesian, shrinkage estimation, small-variance priors

## Abstract

This article describes some potential uses of Bayesian estimation for time-series and panel data models by incorporating information from prior probabilities (i.e., priors) in addition to observed data. Drawing on econometrics and other literatures we illustrate the use of informative “shrinkage” or “small variance” priors (including so-called “Minnesota priors”) while extending prior work on the general cross-lagged panel model (GCLM). Using a panel dataset of national income and subjective well-being (SWB) we describe three key benefits of these priors. First, they shrink parameter estimates toward zero or toward each other for time-varying parameters, which lends additional support for an income → SWB effect that is not supported with maximum likelihood (ML). This is useful because, second, these priors increase model parsimony and the stability of estimates (keeping them within more reasonable bounds) and thus improve out-of-sample predictions and interpretability, which means estimated effect should also be more trustworthy than under ML. Third, these priors allow estimating otherwise under-identified models under ML, allowing higher-order lagged effects and time-varying parameters that are otherwise impossible to estimate using observed data alone. In conclusion we note some of the responsibilities that come with the use of priors which, departing from typical commentaries on their scientific applications, we describe as involving reflection on how best to apply modeling tools to address matters of worldly concern.

## From Data to Causes III: Bayesian Priors for General Cross-Lagged Panel Data Models (GCLM)

Panel data models track multiple independent units *N* over multiple occasions of measurement *T* with parameters typically estimated by frequentist methods (e.g., Arellano, 2003; Baltagi, 2013; Little, 2013; Allison, 2014; Hsiao, 2014; Hamaker et al., 2015). This approach to causal inference was recently illustrated by Zyphur et al. (2020a,b), showing the benefits of a general cross-lagged panel model (GCLM) specified as a structural equation model (SEM) and estimated by maximum likelihood. However, moving away from such frequentist estimators, time-series, and panel data models can be extended to allow additional flexibility in data and model structures, thereby enhancing the range of applications and practical usefulness of models such as the GCLM.

In the current article we do this by showing how Bayesian estimation and inference can expand the range of available model specifications because Bayesian approaches allow including information from prior probabilities (i.e., priors) as well as observed data when estimating parameters (for general discussions see Gill, 2008; Gelman et al., 2014). Prior probabilities can be specified in various ways when estimating panel data models (e.g., Schuurman et al., 2016) including weakly informative priors to improve the stability of estimates (keeping them within more reasonable bounds; Lüdtke et al., 2018), but here we illustrate the use of informative “small variance” or “shrinkage” priors for parameters and/or parameter differences using an approach that follows from existing work (see Muthén and Asparouhov, 2012; Asparouhov et al., 2015; Zyphur and Oswald, 2015). This approach to informative priors “shrinks” parameter estimates toward zero or toward each other while allowing estimates to deviate from these priors as a function of observed data.

In this article we endeavor to show how, in the context of panel data models, such priors have many benefits, helping to solve the problem of “how to build models that are flexible enough to be empirically relevant … but not so flexible as to be seriously over-parameterized” (Koop and Korobilis, 2010, p. 269). In brief, these priors allow many parameters to be estimated while at the same time minimizing model complexity, shrinking parameter estimates toward zero, and/or toward each other by inducing a strong positive correlation among parameters (i.e., reducing parameter differences; Korobilis, 2013). Two key benefits of this prior specification and of Bayesian estimation and inference more generally are as follows.

First, the priors increase generalizability by reducing variance in a classic bias-variance trade-off, which is important for practically applying results from panel data models by reducing overfitting (Korobilis, 2013). Second, they allow estimating models that are under-identified in frequentist approaches due to limited *T* and/or *N*, such as when estimating time-varying unit effects and multiple lagged effects (see Canova, 2007; Koop and Korobilis, 2010; Canova and Ciccarelli, 2013; Giannone et al., 2015). By using informative priors, under-identified parameters need not be strictly constrained to zero or equality over time as would be required with frequentist estimators, thus allowing model results to be more sensitive to observed data patterns when compared to models that constrain parameters to zero or equality over time.

In what follows, we illustrate these benefits by first reviewing the GCLM and its identification in SEM under frequentist estimators. We then describe Bayesian estimation and inference, focusing on the benefits of small-variance priors. Using Gallup World Poll data from Diener et al. (2013) used in Zyphur et al.’s articles, we then fit various models to illustrate the benefits of our Bayesian approach. In so doing, we support different conclusions than the original two articles on the GCLM, which revealed no causal effects among income and subjective well-being (SWB). With a Bayesian approach, we show a positive short-run and long-run effect of income on SWB, but not the reverse. We conclude with brief thoughts on panel data models, including the importance of using them to study processes that are of serious worldly concern. Before continuing we emphasize that our effort here is to illustrate some of the logic and potential uses of prior probabilities for time-series and panel data models, rather than provide a comprehensive overview of priors in longitudinal data models. Other work on priors, sensitivity analyses, and reporting standards exists and we advise interested authors to further explore these topics (e.g., Depaoli and van de Schoot, 2017; Smid et al., 2020), including specifically in the domain of panel data models similar to the GCLM (Lüdtke et al., 2018).

## The General Cross-Lagged Panel Model (GCLM)

The GCLM is specified for a unit *i* at an occasion *t* with two variables *x*_*i,t*_ and *y*_*i,t*_ (for additional insight see Zyphur et al., 2020a,b). Parenthetical superscripts (*x*) and (*y*) indicate the equation in which a coefficient belongs; subscripts *x* and *y* indicate the predictor with which a coefficient is associated; and *h* indicates a lag or lead, such as *y*_*i,t–h*_. With this notation, the general model is shown as follows (for *t* > 1):

(1) $$x_{i,t}=\alpha_{t}^{\left(x\right)}+\lambda_{t}^{\left(x\right)}\,\eta_{i}^{\left(x\right)}+\beta_{x\,1}^{\left(x\right)}\,x_{i,t-1}+\delta_{x\,1}^{\left(x\right)}\,u_{i,t-1}^{\left(x\right)}+\beta_{y\,1}^{\left(x\right)}\,y_{i,t-1}+\delta_{y\,1}^{\left(x\right)}\,u_{i,t-1}^{\left(y\right)}+u_{i,t}^{\left(x\right)}$$

(2) $$y_{i,t}=\alpha_{t}^{\left(y\right)}+\lambda_{t}^{\left(y\right)}\,\eta_{i}^{\left(y\right)}+\beta_{y\,1}^{\left(y\right)}\,y_{i,t-1}+\delta_{y\,1}^{\left(y\right)}\,u_{i,t-1}^{\left(y\right)}+\beta_{x\,1}^{\left(y\right)}\,x_{i,t-1}+\delta_{x\,1}^{\left(y\right)}\,u_{i,t-1}^{\left(x\right)}+u_{i,t}^{\left(y\right)}$$

wherein *u*_*i,t*_ is an impulse capturing random events that are meant to mimic random assignment to levels of a variable, with variance ψ_*u_t*_ and contemporaneous covariance or “co-movement”$\psi_{u_{t}}^{\left(x\,y\right)}$; α_*t*_is an occasion effect at a time *t*; η_*i*_is a unit effect capturing stable factors over time, with$\eta_{i}^{\left(x\right)}∼N\,\left(0,\psi_{\eta }^{\left(x\right)}\right)$, $\eta_{i}^{\left(y\right)}∼N\,\left(0,\psi_{\eta }^{\left(y\right)}\right)$, and covariance$\psi_{\eta }^{\left(x\,y\right)}$; λ_*t*_ is a time-varying unit effect; $\beta_{x\,1}^{\left(x\right)}$ and $\beta_{y\,1}^{\left(y\right)}$ are autoregressive (AR) effects of past impulses on the same variable (with coefficients on lagged predictors taking a form $\beta_{y\,h}^{\left(y\right)}$, wherein *h* is the lag); $\delta_{x\,1}^{\left(x\right)}$ and $\delta_{y\,1}^{\left(y\right)}$ are moving average or MA effects of past impulses on the same variable; $\beta_{y\,1}^{\left(x\right)}$ and $\beta_{x\,1}^{\left(y\right)}$ are cross-lagged or CL effects of past impulses on another variable; and $\delta_{y\,1}^{\left(x\right)}$ and $\delta_{x\,1}^{\left(y\right)}$ are cross-lagged moving average or CLMA effects of past impulses among different variables^1^. With this logic, we interpret at least three kinds of effects: (1) total effects of a variable on itself combine AR and MA terms to show the short-run persistence of impulses [e.g., $\beta_{y\,1}^{\left(y\right)}+\delta_{y\,1}^{\left(y\right)}$] such that a process is more mean-reverting as these terms tend towards zero; (2) Granger-causal effects of impulses that combine all CL and CLMA terms to show short-run or direct effects among different variables over time [e.g., $\beta_{x\,1}^{\left(y\right)}+\delta_{x\,1}^{\left(y\right)}$]; and (3) impulse responses map the change in a system across all parameters due to an impulse [e.g., a change along $u_{i,t}^{\left(y\right)}$], showing long-run or total effects of an impulse across all variables in a system over time (see Zyphur et al., 2020a).

We map this general model structure onto the following SEM:

(3) $${\text{y}}_{i}=\Lambda \,\eta_{i}$$

(4) $$\eta_{i}=\alpha +\text{B}\,\eta_{i}+\zeta_{i}$$

with all terms as follows for an AR(1)MA(1)CL(1)CLMA(1) model and a single unit effect for each of *k* observed variables at *T* occasions: **y**_*i*_is a *kT* length vector of observed variables; **Λ** is a *kT*×(2*kT* + *k*) matrix, mapping *kT* observed variables onto *kT* latent analogs; η_*i*_ is a *2kT+k* length vector, with *kT* terms mapped to **y**_*i*_, *kT* impulses, and *k* unit effects; α is a *2kT+k* length vector with *kT* occasion effects only; **B** is a (2*kT* + *k*)×(2*kT* + *k*) matrix with *kT* unities to map *kT* observed variables to *kT* impulses, *kT* time-varying unit effects, 2*k* AR and MA terms, and 2*k*(*k*−1) CL and CLMA terms; and ζ_*i*_ is a *2kT+k* length vector with covariance matrix **Ψ** containing *k* unit effect variances, *k*(*k*−1)/2 unit effect covariances, *kT* impulse variances, and *kT*(*k*−1)/2 co-movements.

As with time-series and panel data models in general, the GCLM requires choosing different numbers of unit effects and the following lag orders: *p* lags in an AR(*p*) model; *q* lags in an MA(*q*) model; *c* lags in an CL(*c*) model; *l* lags in an CLMA(*l*) model. Substantive and statistical checking should inform these choices, with an emphasis on conservative models that balance theory and contextual knowledge with model fit (Armstrong et al., 2015; Green and Armstrong, 2015). In Zyphur et al. (2020a) this was done by modeling income *x*_*i,t*_ and SWB *y*_*i,t*_ for *N* = 135 countries and *T* = 6 years from 2006 to 2011 (see Diener et al., 2013). After substantive and statistical checking, an AR(1)MA(2)CL(1)CLMA(1) model was chosen for income *x*_*i,t*_ (adding a higher-order MA term $\delta_{x\,2}^{\left(x\right)}\,u_{i,t-2}^{\left(x\right)}$ to Eq. 1), and an AR(1)MA(1)CL(1)CLMA(1) model was chosen for SWB *y*_*i,t*_ (fitting with Eq. 2).

**FIGURE 1:**
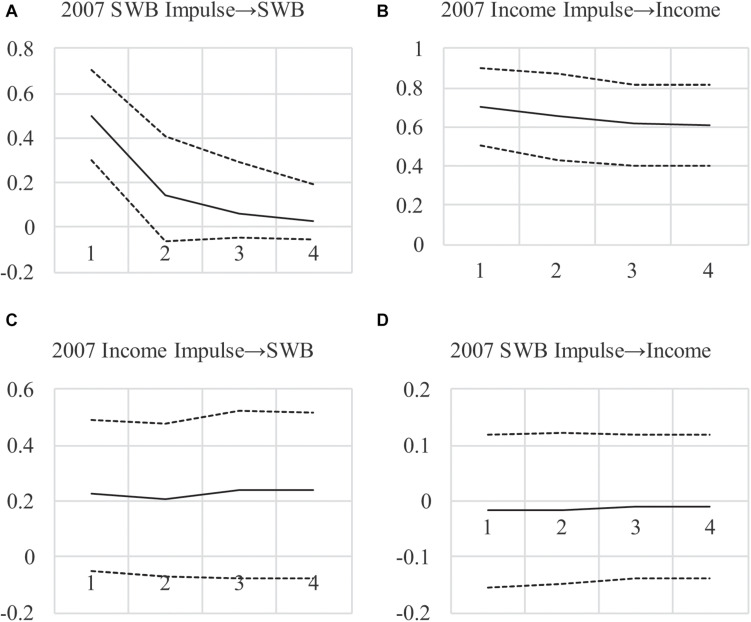
**(A–D)** Impulse Response Functions for AR(1)MA(2) Model Under Maximum-Likelihood. Impulses begin in 2007, showing the effect of a 1-unit impulse in 2007 over the next 4 years with 95% confident intervals.

Descriptive statistics are in Zyphur et al. (2020a) and results are in Table 1 as the maximum-likelihood or “ML” model these authors estimated. Table 2 shows Granger causality tests from the four steps discussed by these authors, with AIC and BIC values showing that eliminating CL and CLMA effects improves model fit (by decreasing AIC and BIC values, indicating better model quality as a trade-off between fit and parsimony). This fails to support any form of Granger causality using the logic from Zyphur et al. (2020a). Finally, impulse responses in Figures 1A–D show very weak support for long-run effects with *CI*s that include zero, and an unexpected negative SWB → income effect. In sum, these results are counter to those originally presented by Diener et al. (2013), who found a positive income → SWB effect as well as a positive SWBincome effect, which Zyphur et al. (2020a) proposed was likely due to failing to control for unit effects $\eta_{i}^{\left(x\right)}$ and $\eta_{i}^{\left(y\right)}$ (and their covariance $\psi_{\eta }^{\left(x\,y\right)}$).

**TABLE 1:** Model results.

Parameters	Estimates (*SE*s or posterior *SD*s) (Ranges for time-varying parameters)		
	
	ML	Bayes 1	Bayes 2
**SWB → SWB AR/MA Terms $\beta_{y\,1}^{\left(y\right)}$ and $\delta_{y\,1}^{\left(y\right)}$**			
$\beta_{y\,1}^{\left(y\right)}$	0.39 (0.36)	0.34 (0.21) [0.30, 0.39]	0.34 (0.21) [0.29, 0.39]
$\delta_{y\,1}^{\left(y\right)}$	0.19 (0.32)	0.15 (0.19) [0.08, 0.22]	0.16 (0.19) [0.08, 0.23]
$\beta_{y\,1}^{\left(y\right)}+\delta_{y\,1}^{\left(y\right)}$	0.58* (0.09)	0.49* (0.08) [0.38, 0.62]	0.49* (0.08) [0.38, 0.62]
**Income → Income AR/MA terms $\beta_{x\,1}^{\left(x\right)}$ and** $\delta_{x\,1}^{\left(x\right)}$			
$\beta_{x\,1}^{\left(x\right)}$	0.96* (0.13)	0.97* (0.03) [0.94, 1.0]	0.97* (0.03) [0.94, 1.0]
$\delta_{x\,1}^{\left(x\right)}$	−0.33 (0.25)	−0.27* (0.07) [−0.30, −0.21]	−0.26* (0.07) [0.30, −0.21]
$\delta_{x\,2}^{\left(x\right)}$	0.06 (0.09)	0.02 (0.06) [−0.03, 0.08]	0.01 (0.04) [−0.07, 0.11]
$\delta_{x.}^{\left(x\right)}$	−0.27 (0.19)	−0.25* (0.10) [−0.32, −0.13]	−0.26* (0.08) [−0.35, −0.10]
$\beta_{x\,1}^{\left(x\right)}+\delta_{x.}^{\left(x\right)}$	0.69* (0.20)	0.72* (0.08) [0.62, 0.87]	0.72* (0.07) [0.59, 0.90]
**Income → Subjective well-being CL/CLMA terms $\beta_{x\,1}^{\left(y\right)}$ and $\delta_{x\,1}^{\left(y\right)}$**			
$\beta_{x\,1}^{\left(y\right)}$	0.13 (0.32)	0.23 (0.20) [0.19, 0.26]	0.24 (0.20) [0.20, 0.26]
$\delta_{x\,1}^{\left(y\right)}$	0.01 (0.25)	−0.03 (0.19) [−0.07, 0.01]	−0.03 (0.19) [−0.08, 0.002]
$\beta_{x\,1}^{\left(y\right)}+\delta_{x\,1}^{\left(y\right)}$	0.14 (0.16)	0.22 (0.12) [0.12, 0.25]	0.22 (0.12) [0.12, 0.24]
**Subjective well-being → Income CL/CLMA terms $\beta_{y\,1}^{\left(x\right)}$ and $\delta_{y\,1}^{\left(x\right)}$**			
$\beta_{y\,1}^{\left(x\right)}$	−0.10 (0.07)	0.01 (0.03) [−0.001, 0.03]	0.01 (0.02) [−0.001, 0.03]
$\delta_{y\,1}^{\left(x\right)}$	0.08 (0.07)	−0.02 (0.05) [−0.04, 0.01]	−0.02 (0.05) [−0.04, 0.01]
$\beta_{y\,1}^{\left(x\right)}+\delta_{y\,1}^{\left(x\right)}$	−0.02 (0.04)	−0.01 (0.05) [−0.04, 0.01]	−0.01 (0.04) [−0.04, 0.01]
**Co-movement in impulses $\psi_{u_{t}}^{\left(x\,y\right)}$ as correlations**			
$\psi_{u_{1}}^{\left(x\,y\right)}$	0.64 (0.59)	0.87* (0.31)	0.87* (0.30)
$\psi_{u_{2}}^{\left(x\,y\right)}$	0.45* (0.21)	0.44* (0.17)	0.44* (0.17)
$\psi_{u_{3}}^{\left(x\,y\right)}$	0.003 (0.13)	0.05 (0.13)	0.05 (0.13)
$\psi_{u_{4}}^{\left(x\,y\right)}$	−0.02 (0.12)	0.05 (0.13)	0.06 (0.13)
$\psi_{u_{5}}^{\left(x\,y\right)}$	0.32* (0.14)	0.41* (0.11)	0.41* (0.11)
$\psi_{u_{6}}^{\left(x\,y\right)}$	0.11 (0.13)	0.14 (0.11)	0.15 (0.11)
**Unit effect variances $\psi_{\eta }^{\left(y\right)}$ and $\psi_{\eta }^{\left(x\right)}$, and covariance $\psi_{\eta }^{\left(x\,y\right)}$ as a correlation**			
$\psi_{\eta }^{\left(y\right)}$	1.01	1.01	1.01
$\psi_{\eta }^{\left(x\right)}$	0.40	0.37	0.37
$\psi_{\eta }^{\left(x\,y\right)}$	0.96* (0.06)	0.86* (0.18)	0.86* (0.18)
**Time-varying unit effects (“factor loadings”) $\lambda_{t}^{\left(y\right)}$ and $\lambda_{t}^{\left(x\right)}$ as correlations**			
$\lambda_{1}^{\left(y\right)}$	0.96* (0.06)	0.92* (0.10)	0.91* (0.10)
$\lambda_{2}^{\left(y\right)}$	0.48 (0.32)	0.47* (0.18)	0.47* (0.18)
$\lambda_{3}^{\left(y\right)}$	0.48 (0.32)	0.44* (0.17)	0.44* (0.17)
$\lambda_{4}^{\left(y\right)}$	0.46 (0.30)	0.51* (0.18)	0.51* (0.17)
$\lambda_{5}^{\left(y\right)}$	0.52 (0.30)	0.45* (0.17)	0.45* (0.17)
$\lambda_{6}^{\left(y\right)}$	0.46 (0.33)	0.44* (0.18)	0.45* (0.17)
$\lambda_{1}^{\left(x\right)}$	0.73* (0.25)	0.69* (0.19)	0.68* (0.19)
$\lambda_{2}^{\left(x\right)}$	−0.03 (0.22)	−0.01 (0.06)	−0.01 (0.06)
$\lambda_{3}^{\left(x\right)}$	0.15 (0.09)	−0.01 (0.05)	−0.01 (0.05)
$\lambda_{4}^{\left(x\right)}$	0.16* (0.07)	0.02 (0.05)	0.02 (0.05)
$\lambda_{5}^{\left(x\right)}$	0.15* (0.08)	0.01 (0.06)	0.01 (0.06)
$\lambda_{6}^{\left(x\right)}$	0.16* (0.08)	−0.01 (0.05)	−0.01 (0.05)
**Fit indices**			
*k*/*pD*	54	50.69	50.78
*PPP*	–	0.32	0.32
DIC	–	829.23	827.65

ML, maximum likelihood; SWB, subjective well-being; AR, autoregressive; MA, moving average; CL, cross-lagged; CLMA, cross-lagged moving average; BIC, Bayes information criterion; DIC, deviance information criterion; *k/pD*, the number of model parameters, which is exactly *k* under ML and is estimated as *pD* for Bayes models. * p < 0.05.

**TABLE 2:** Granger Causality Tests and Δ*R*^2^.

ML	Bayes 1	Bayes 2
AIC/BIC	DIC	DIC
*Step 1: Derive fit of full model*		
845.94/1002.82	829.23	827.65
*Step 2*: *Constraint all income* → *SWB effects*		
841.86/990.03	835.48	833.85
*Step 3*: *Constrain all SWB* → *Income effects*		
844.08/992.25	820.83	818.71
*Step 4*: *Constraining all CL/CLMA terms*		
842.62/984.98	833.35	831.70

*ML, maximum-likelihood; SWB, subjective well-being; AIC, Akaike’s information criterion; BIC, Bayes information criterion; DIC, Deviance information criterion.*

However, the model chosen by Zyphur et al., was limited by their reliance on a frequentist estimator. Although these estimators are common and inferences based on them may be sound in many cases, estimators such as ML rely on only observed data rather than also incorporating prior information about parameters (van de Schoot et al., 2017). Specifically, time-varying unit effects λ_*t*_η_*i*_ and AR/MA terms rely on *kT*(*T*−1)/2 observed auto-covariances for estimation. On the other hand, unit effect covariances $\psi_{\eta }^{\left(x\,y\right)}$, CL/CLMA terms, and impulse co-movements $\psi_{u_{t}}^{\left(x\,y\right)}$ rely on *k*(*k*−1)*T*^2^/2 observed cross-covariances for estimation. In turn, for an SEM to be identified the number of observed auto- and cross-covariances (associated with *T*) must grow with the number of time-varying unit effects λ_*t*_η_*i*_ and the *p*, *q*, *c*, and *l* lag orders for AR, MA, CL, and CLMA terms (for a general discussion of identification see Bollen, 1989). Also, with many estimated parameters, the *N* required to assure asymptotic assumptions are met for ML also increases. Furthermore, even with large *T* and *N*, some models may not be identified and may violate asymptotic assumptions, such as if AR, MA, CL, and CLMA effects are time-varying, which we can show by modifying Eqs. 1 and 2 with a *t* subscript as follows (for *t* > 1):

(5) $$x_{i,t}=\alpha_{t}^{\left(x\right)}+\lambda_{t}^{\left(x\right)}\,\eta_{i}^{\left(x\right)}+\beta_{x\,1,t}^{\left(x\right)}\,x_{i,t-1}+\delta_{x\,1,t}^{\left(x\right)}\,u_{i,t-1}^{\left(x\right)}+\beta_{y\,1,t}^{\left(x\right)}\,y_{i,t-1}+\delta_{y\,1,t}^{\left(x\right)}\,u_{i,t-1}^{\left(y\right)}+u_{i,t}^{\left(x\right)}$$

(6) $$y_{i,t}=\alpha_{t}^{\left(y\right)}+\lambda_{t}^{\left(y\right)}\,\eta_{i}^{\left(y\right)}+\beta_{y\,1,t}^{\left(y\right)}\,y_{i,t-1}+\delta_{y\,1,t}^{\left(y\right)}\,u_{i,t-1}^{\left(y\right)}+\beta_{x\,1,t}^{\left(y\right)}\,x_{i,t-1}+\delta_{x\,1,t}^{\left(y\right)}\,u_{i,t-1}^{\left(x\right)}+u_{i,t}^{\left(y\right)}$$

This model allows for “regime changes” as changes in effects over time (Stock and Watson, 1996, 2009), which is reasonable given the fact that people, organizations, and entire economies are complex dynamic systems that are always in flux (Williams and Cook, 2016). However, Eqs. 5 and 6 imply that there are now *T-1* unique parameters for *each* AR, MA, CL, and CLMA term, and these proliferate rapidly as *k* increases, such that the total number of time-varying AR, MA, CL, and CLMA effects is (*T*−1)[2*k* + 2*k*(*k*−1)]. For example, with *k* = 4 observed variables and *T* = 10 occasions of measurement, Eqs. 5 and 6 imply a model with 288 β and δ terms, requiring large *N*. Furthermore, this large number of terms is based on lag orders that are limited to the simplest *p* = *q* = *c* = *l* = 1 case, which will not always hold in practice and, when it does not, will put substantial requirements on observed data and the estimates derived from them.

Clearly, for GCLMs like that in Eqs. 5 and 6 and for panel data models more generally, parameter identification and overfitting as well as meeting ML assumptions may be difficult (Lüdtke et al., 2018), especially as lag orders and the number of unit effects grow. Due to this problem, parameter estimates—and therefore Granger causality tests and impulse responses—may have reduced generalizability and the number of parameters that can be estimated are limited by *N* and *T*. This is unfortunate for many reasons, such as difficulty in supporting hypotheses due to moderate *N*. Also, ironically, the parameter restrictions required to achieve model identification run counter to the impetus for panel data models like ours, which is partly to overcome the “incredible” identifying assumptions typically found in regression models (see Sims, 1980, 1986). In order to provide a solution to these problems, we now describe a Bayesian approach to estimation and inference.

## Bayesian Estimation and Inference

There are two key differences between Bayesian and frequentist estimation. The first and perhaps primary difference is that whereas frequentist probabilities apply to data (or events), Bayesian probabilities apply to parameters (or hypotheses; Zyphur and Oswald, 2015). The implication is that instead of representing relative frequencies, probabilities represent degrees of belief or knowledge (Howson and Urbach, 2006). The classic idea is that Bayesian probabilities are meant to be inductive, allowing direct probabilistic inferences about parameters in a model θ given observed data *Y* (Hacking, 2001; Jaynes, 2003). With this orientation, Bayesian estimation and inference are done in order to represent degrees of uncertainty around parameters, measured by a “posterior” probability distribution *f*(θ|*Y*). The mean, median, or mode of this distribution is used to describe specific parameter point estimates and variance in the distribution is used to describe uncertainty in parameters for hypothesis testing. For example, the *SD* of a parameter distribution can be used to approximate a frequentist *SE* for the computation of Bayesian *p*-values (for discussion, see Muthén and Asparouhov, 2012; Zyphur and Oswald, 2015). In all cases, posterior distributions are meant to represent knowledge or beliefs about parameters, with hypothesis tests serving to inform knowledge or beliefs about parameters based on model results.

The second difference between frequentist and Bayesian methods is how such results are derived, which is to say how a posterior distribution *f*(θ|*Y*) is estimated. Unlike frequentist estimation, Bayesian estimators must directly incorporate two sources of information to estimate parameters in a model_θ_: prior probabilities of parameters *f*(θ) that serve to indicate the knowledge or beliefs about parameters before estimation; and the probability of observed data *Y* given parameter estimates *f*(*Y*|θ), which can be understood as a likelihood. The result is posterior probabilities *f*(θ|*Y*), which are then used for inference. The proportional relation (∝) among these terms can be shown as follows (see Muthén and Asparouhov, 2012):

(7) f⁢(θ|Y)∝f⁢(θ)⁢f⁢(Y|θ)

wherein model results *f*(θ|*Y*) are derived based on both information in the priors *f*(θ) and the data *Y* in the form of the model likelihood *f*(*Y*|θ).

The result of this logic is that Bayesian estimators are justified based on the degree to which they satisfy the rule in Eq. 7, which is designed to be a logically consistent system for updating prior knowledge or beliefs with additional data (Zyphur and Oswald, 2015). This is very much unlike frequentist estimators, which are justified based on asymptotic theories that describe how estimators perform when, for example, a sample size grows to infinity and/or a study is conducted an infinite number of times. One result of this difference between Bayesian and frequentist logics is that frequentist estimators like ML satisfy assumptions only as *N*→∞, which creates problems for SEM with many parameters and small *N* (Anderson and Gerbing, 1984; MacCallum et al., 1996). Conversely, because Bayesian estimation requires only that the rule in Eq. 7 be followed, models with many parameters and small *N* are not problematic apart from the way that small *N* exacts an appropriate toll by increasing levels of uncertainty in *f*(θ|*Y*) (rather than also violating assumptions about the estimator in relation to *N*; for insight into the importance of priors in such cases see Smid et al., 2020). The point is that as long as estimation follows the rule in Eq. 7, then a sample size *N* is always appropriate even if it makes reducing uncertainty in a posterior distribution *f*(θ|*Y*) difficult.

Given the focus on Eq. 7, a key question to answer for a Bayesian approach is how to choose prior probabilities for model parameters in *f*(θ). Typically, “uninformative” or “diffuse” priors are used for*f*(θ) in an attempt to eliminate their influence on posteriors *f*(θ|*Y*) (Gelman et al., 2014). The point of these priors can be conceptualized as “flattening” probability (i.e., “leveling” belief or knowledge) across the range of possible parameter values in θ. This is akin to being agnostic about specific parameter values (i.e., having no strong prior knowledge or beliefs), which is meant to result in reducing the influence of priors *f*(θ) during estimation. In turn, such priors produce strong agreement among Bayesian and frequentist estimates as *N* increases, which is sensible because as priors’ influence decreases, posteriors are increasingly dominated by the likelihood *f*(*Y*|θ) that many frequentist methods maximize (the reader can see this by conceptually by removing the prior from Eq. 7). In turn, statistical modeling programs such as Mplus often use various kinds of diffuse priors by default, such as a prior for a regression slope with a variance that is, practically speaking, infinity, such as β∼*N*(0,10^10^) (Asparouhov and Muthén, 2010; Muthén, 2010). The reader can intuit how this prior is uninformative by recognizing that the mean of the distribution 0 has virtually no greater probability than a value of 100 for β, because β∼*N*(0,10^10^) implies an extremely flat probability distribution (i.e., approximately equal belief or knowledge for any specific value of β).

Conversely, priors become informative and increasingly influential as they become increasingly dense around specific parameter values, such as a small-variance prior for a regression slope β∼*N*(0,0.01) (Muthén and Asparouhov, 2012; Zyphur and Oswald, 2015). In this case, the density of the prior distribution is high around the value 0, and during estimation this pulls estimates of β toward 0 (Gill, 2008; Gelman et al., 2014). Thus, informative priors that favor null parameter values effectively “shrink” parameter estimates toward 0, which is useful because this increases generalizability by reducing the tendency to overfit model estimates to an observed dataset (McNeish, 2015). As Giannone et al. (2015) note, priors such as these “are successful because they effectively reduce the estimation error while generating only relatively small biases in the estimates of the parameters” (p. 436). Of course it is notable that alternative small-variance priors can be chosen—as we note further below with references to relevant work that the reader may consult—our choice of small-variance priors here follows from existing work using these in the psychology and organizational literature (see Muthén and Asparouhov, 2012; Zyphur and Oswald, 2015).

Furthermore, because Bayesian estimation relies on prior probabilities *f*(θ) and the likelihood *f*(*Y*|θ), priors behave more like observed data when they favor specific parameter values—whatever these might be. By this we mean that in a model with small-variance priors, parameters will be identified as a function of the information in observed data *and* the priors, so that even if there is insufficient information in a dataset to identify a parameter, the small-variance prior may serve to help identification. This can be understood by considering that as priors *f*(θ) become more informative, this is akin to a reduction in the number of parameters that are freely estimated in a Bayesian model (symbolized as *pD*). In turn, a diffuse prior such as β∼*N*(0,10^10^) offers little help in identifying estimates of β without sufficient information in the likelihood *f*(*Y*|θ) to do so. On the other hand, a small-variance prior such as β∼*N*(0,0.01) may allow estimating β even when there is insufficient information in the model likelihood to do so (e.g., if a likelihood is relatively “flat” across a range of values for β; Asparouhov et al., 2015). This is because a model with a small-variance prior for the β does not “freely” estimate it in a frequentist sense, but instead combines the prior β∼*N*(0,0.01) with the data *Y* in the form of the likelihood *f*(*Y*|θ).

In sum, informative priors, such as small-variance priors, are useful because they can shrink estimates to avoid overfitting, thereby increasing generalizability, while at the same time helping to identify parameters that otherwise may not be estimable due to insufficient information in a dataset *Y*. Furthermore, these priors can serve to operationalize prior knowledge or beliefs about parameters, while allowing data to update the priors to produce results that combine these two sources of information. As previously noted, this is consistent with the interest of an informal Bayesian who seeks to use panel data models to change knowledge or beliefs about the ways in which variables are causally related over time (Granger, 1980).

### Priors for Time-Series and Panel Data Models

Due to their ability to address overfitting and non-identified parameters, informative priors have become popular in time-series and panel data modeling, particularly in a vector autoregressive or VAR framework (for discussions, see Canova, 2007; Koop and Korobilis, 2010; Giannone et al., 2015). To illustrate this, the approach we use here relies on small-variance priors for parameters as well as parameter differences for time-varying terms. As examples, consider that higher-order lags may be shrunk toward zero, such as a second-order MA effect: $\delta_{y\,2}^{\left(y\right)}∼N\,\left(0,0.01\right)$; or, differences in time-varying parameters may be shrunk toward each other, such as AR effects at different occasions: $\left(\beta_{x\,1,t}^{\left(x\right)}-\beta_{x\,1,t+1}^{\left(x\right)}\right)∼N\,\left(0,0.01\right)$. Although the former approach may be somewhat familiar (especially in the econometric VAR community), the latter approach is more novel and is designed for cases wherein similar parameters are expected to have small differences. To understand priors such as $\left(\beta_{x\,1,t}^{\left(x\right)}-\beta_{x\,1,t+1}^{\left(x\right)}\right)∼N\,\left(0,0.01\right)$, it may be useful to connect this to terms associated with an SEM (e.g., Eqs. 3 and 4). Specifically, a prior distribution for regression terms in a matrix **B**, or *f*(**B**), may be parameterized as *f*(**B**)∼*MVN*(0,**Ψ_B_**), with the covariance matrix **Ψ_B_** having diagonal elements that imply a diffuse prior distribution (e.g., 1000) and off-diagonal elements that imply large covariances among the parameters (e.g., 999.95). Taken together, the large on-diagonal values imply that, on average, parameter values will be largely driven by the data *Y*, but the large off-diagonal values operationalize a prior expectation of very small parameter differences, thus shrinking parameters toward each other during estimation (*without* also shrinking them toward zero).

This approach with small-variance priors is a simplification of others, such as state-space models with hierarchical priors (see Koop and Korobilis, 2010; Korobilis, 2013). Although these other methods can be approximated using our approach in various ways (for insight see Chow et al., 2010), our goal is not to extend these other methods but instead to provide an introduction to using small-variance priors for panel data models in SEM within a very user-friendly framework. For this our Mplus input and output are available in Supplementary Material with the required data from Zyphur et al.’s online materials so that the reader can freely experiment with priors in GCLMs (notably R users can convert our basic GCLM code into Lavaan using the R program Mplus2lavaan, available here: https://rdrr.io/cran/lavaan/man/mplus2lavaan.html).

The interested reader may also want to examine more technical on the choice of small-variance priors after exploring our article (e.g., Canova, 2007; Canova and Ciccarelli, 2013), especially that which covers the level of prior informativeness in the form of prior variances (e.g., Giannone et al., 2015). Related work also exists in psychology showing that weakly informative priors can help stabilize model parameters in models similar to the GCLM (Lüdtke et al., 2018), which as we show offers important insights that helps motivate some small-variance prior specifications. For pedagogical purposes, we set prior variances at 0.01 (i.e., a prior *SD* of 0.1) in order to express somewhat strong prior expectations that parameters are close to the mean and to be consistent with existing work on small-variance priors (Muthén and Asparouhov, 2012; Zyphur and Oswald, 2015), but in practice researchers may use sensitivity analyses to examine informativeness or they may use automated techniques to determine prior variances (e.g., Giannone et al., 2015).

## The GCLM With Small-Variance Priors

In order to show how a Bayesian approach to estimation and inference can benefit time-series and panel data models (or other models), we now modify the GCLM presented previously and we alter the way it has been estimated by using small-variance priors. We begin with time-varying parameters that incorporate small-variance priors for differences in parameter estimates over time (sometimes called time-varying effects models or TVEMs) and then we proceed to a more traditional form of small-variance “Minnesota” prior for higher-order lags in panel data models—named for the location of the central bank and economists who pioneered the approach.

### Time-Varying Parameters

Our general panel data model from Eqs. 1 and 2 can be usefully extended by allowing time-varying AR, MA, CL, and CLMA effects, which we show as follows (for *t* > 2):

(8) $$x_{i,t}=\alpha_{t}^{\left(x\right)}+\lambda_{t}^{\left(x\right)}\,\eta_{i}^{\left(x\right)}+\beta_{x\,1,t}^{\left(x\right)}\,x_{i,t-1}+\delta_{x\,1,t}^{\left(x\right)}\,u_{i,t-1}^{\left(x\right)}+\delta_{x\,2,t}^{\left(x\right)}\,u_{i,t-2}^{\left(x\right)}+\beta_{y\,1,t}^{\left(x\right)}\,y_{i,t-1}+\delta_{y\,1,t}^{\left(x\right)}\,u_{i,t-1}^{\left(y\right)}+u_{i,t}^{\left(x\right)}$$

(9) $$y_{i,t}=\alpha_{t}^{\left(y\right)}+\lambda_{t}^{\left(y\right)}\,\eta_{i}^{\left(y\right)}+\beta_{y\,1,t}^{\left(y\right)}\,y_{i,t-1}+\delta_{y\,1,t}^{\left(y\right)}\,u_{i,t-1}^{\left(y\right)}+\beta_{x\,1,t}^{\left(y\right)}\,x_{i,t-1}+\delta_{x\,1,t}^{\left(y\right)}\,u_{i,t-1}^{\left(x\right)}+u_{i,t}^{\left(y\right)}$$

wherein all terms are as described previously. This kind of specification is important because researchers have found that some of the greatest improvements in fit and prediction come from allowing time-varying parameters (a type of non-stationarity; Sims and Zha, 2006). However, in our case of *k* = 2 and *T* = 6, this model is not identified with a frequentist estimator because of the many time-varying terms. For example, income *x*_*i,t*_ has 19 parameters that rely on only 15 auto-covariances for estimation: five time-varying unit effects λ_*t*_; one unit effect variance$\psi_{\eta }^{\left(x\right)}$$\psi_{\eta }^{\left(x\right)}$; five AR terms; and eight MA terms. Also, even the SWB variable with only an MA(1) specification has 16 unique parameters that rely on 15 auto-covariances, meaning the model is under-identified for both *x* and *y*. Yet, even if the model were identified, the abundance of parameters might overfit the data, producing results that are not as generalizable—a problem that frequentist estimators can produce in panel data models like Eqs. 8 and 9. Furthermore, given our modest sample size *N* = 135, estimating so many parameters calls into question the asymptotic justification for ML in relation to the number of parameters estimated.

In order to increase model parsimony and identify the model while at the same time helping to address asymptotic concerns related to the ML estimator used in Zyphur et al. (2020a), we take a Bayesian approach with small-variance priors for *differences* in AR, MA, CL, and CLMA terms, with priors as follows (for *t* > 1) to allow differences in parameters over time by “shrinking” these differences (i.e., by helping parameters remain similar over time):

$$\mathrm{AR}\,\mathrm{effects}\,\mathrm{for}\,\mathrm{income}:\left(\beta_{x\,1,t}^{\left(x\right)}-\beta_{x\,1,t+1}^{\left(x\right)}\right)∼N\,\left(0,0.01\right)$$

$$\mathrm{MA}\,\mathrm{effects}\,\left(\mathrm{first}-\mathrm{order}\right)\,\mathrm{for}\,\mathrm{income}:\left(\delta_{x\,1,t}^{\left(x\right)}-\delta_{x\,1,t+1}^{\left(x\right)}\right)∼$$

 ⁢N⁢(0,0.01)

$$\mathrm{MA}\,\mathrm{effects}\,\left(\mathrm{second}-\mathrm{order}\right)\,\mathrm{for}\,\mathrm{income}:\left(\delta_{x\,2,t}^{\left(x\right)}-\delta_{x\,2,t+1}^{\left(x\right)}\right)∼$$

 ⁢N⁢(0,0.01)

$$\mathrm{CL}\,\mathrm{effects}\,\mathrm{for}\,\mathrm{income}:\left(\beta_{y\,1,t}^{\left(x\right)}-\beta_{y\,1,t+1}^{\left(x\right)}\right)∼N\,\left(0,0.01\right)$$

$$\mathrm{CLMA}\,\mathrm{effects}\,\mathrm{for}\,\mathrm{income}:\left(\delta_{y\,1,t}^{\left(x\right)}-\delta_{y\,1,t+1}^{\left(x\right)}\right)∼N\,\left(0,0.01\right)$$

$$\mathrm{AR}\,\mathrm{effects}\,\mathrm{for}\,\mathrm{SWB}:\left(\beta_{y\,1,t}^{\left(y\right)}-\beta_{y\,1,t+1}^{\left(y\right)}\right)∼N\,\left(0,0.01\right)$$

$$\mathrm{MA}\,\mathrm{effects}\,\mathrm{for}\,\mathrm{SWB}:\left(\delta_{y\,1,t}^{\left(y\right)}-\delta_{y\,1,t+1}^{\left(y\right)}\right)∼N\,\left(0,0.01\right)$$

$$\mathrm{CL}\,\mathrm{effects}\,\mathrm{for}\,\mathrm{SWB}:\left(\beta_{x\,1,t}^{\left(y\right)}-\beta_{x\,1,t+1}^{\left(y\right)}\right)∼N\,\left(0,0.01\right)$$

$$\mathrm{CLMA}\,\mathrm{effects}\,\mathrm{for}\,\mathrm{SWB}:\left(\delta_{x\,1,t}^{\left(y\right)}-\delta_{x\,1,t+1}^{\left(y\right)}\right)∼N\,\left(0,0.01\right)$$

Where in all terms are as above and the expected differences in each set of parameters are set to a small value. This prior specification implies that the GCLM under ML is not nested within this model with time-varying lagged effects—although it still provides an interesting opportunity to compare results. Specifically, with a mean of 0 and a variance of 0.01, these normally distributed priors set a roughly 68% probability that the parameters are within ++/−. One of each other over time. This is akin to relatively strong prior beliefs that the parameters are similar over time, which can be understood in relation to a prior *f*(**B**)∼*MVN*(0,**Ψ_B_**), with the covariance matrix **Ψ_B_** having large diagonal and off-diagonal elements—implying diffuse priors while at the same time imposing an expectation of similar parameter values over time. Conveniently, observed data will test the veracity of this expectation by pulling posteriors away from these priors if this is warranted by the data (Muthén and Asparouhov, 2012).

To continue, we can increase model parsimony further by setting small-variance priors for time-varying unit effects, which we illustrate in two ways. First, recall that SWB is often highly stable (see Easterlin, 1995, 2001; Diener and Lucas, 1999; Clark et al., 2008). Indeed, in psychology, it is common to assume a form of mean-stationarity for η_*i*_ by setting λ_*t*_≡1 (e.g., Hamaker et al., 2015). This assumption of constant effects is so common that it is the default for multilevel models and most fixed-effects approaches (see Nezlek, 2012a,b; Allison, 2014; Hoffman, 2015). Our results in Table 1 for the ML model support this, showing similar effects for $\lambda_{t}^{\left(y\right)}$ over *T*. Therefore, we set the following small-variance priors to operationalize an expectation of mean-stationarity for $\eta_{i}^{\left(y\right)}$ (for *t*> 1): $\left(\lambda_{t}^{\left(y\right)}-\lambda_{t+1}^{\left(y\right)}\right)∼N\,\left(0,.01\right)$.

This kind of prior specification—operationalizing theory and past findings—is in the spirit of Minnesota priors (see Koop and Korobilis, 2010). In this tradition, econometricians often assume small if any unit effects for variables like income (Canova and Ciccarelli, 2013). Instead, trends are often treated as stochastic rather than deterministic—as noted in Zyphur et al. (2020b)—which is supported by results in Table 1 for the ML model, showing weak time-varying effects $\lambda_{t}^{\left(x\right)}$. Conveniently, Bayesian priors allow a model that incorporates time-varying unit effects but simultaneously bets against them, so to speak. To put this into practice, we use a prior that assumes no unit effects (for *t* > 1) $\lambda_{t}^{\left(x\right)}∼N\,\left(0,0.01\right)$. This prior has multiple benefits: it shrinks unit effects toward zero; it reduces the number of parameters that are freely estimated; and it allows unit effects to manifest in posteriors as a function of the observed data—in part by leaving income’s unit effect variance $\psi_{\eta }^{\left(x\right)}$ unrestricted^2^.

Furthermore, these prior specifications on the factor loadings of the latent unit effects help to resolve a dilemma that other researchers may experience when using a relatively small sample (here *N* = 135) in the presence of modest unit effects variances (for an overview and relevant simulations see Lüdtke et al., 2018). Specifically, the default non-noninformative or diffuse priors in Mplus can cause estimation problems with unit effect variances and their factor loadings, which we encountered with variances tending to zero and loadings that were incredibly large when estimating the GCLM with a Bayes estimator and the default priors in Mplus (we omit results but the reader can find them in our online materials in the file “AR(1)MA(2) (Step 1, Full Model) Bayes.out”). One solution to this problem is imposing a mean-stability assumption by restricting the factor loadings to equality over time (after the *t* = 1 occasion, which resolves the problem with the parameter estimates as shown in the file “AR(1)MA(2) (Step 1, Full Model) Bayes_mean stability.out”). However, the small-variance priors we describe here allow avoiding the mean-stability assumption while also stabilizing the variance and factor loadings estimates.

In sum, the above combination of small-variance priors minimizes model complexity due to time-varying parameters while at the same time allowing the estimation of all parameters even when they are not identified with frequentist estimators or because of other estimation problems. Using a Bayes approach, we estimate the model in Eqs. 8 and 9 with the above priors using a Markov Chain Monte Carlo (MCMC) method with a Gibbs sampler in Mplus. For this and other models that follow, estimation is done with at least 10,000 iterations in two chains—these were thinned by retaining every 50th estimate (for a total of 500,000 iterations) to assure convergence within the 10,000 estimates and eliminate autocorrelation across the iterations.

Convergence is checked by examining the quality of chain mixing with the *estimated* or *potential scale reduction* (*PSR*) factor, with values of 1.05 or less typically used as a cut-off (see Gill, 2008, pp. 478–482; Asparouhov and Muthén, 2010). We also use Kolmogorov-Smirnov tests that compute *p*-values for parameter differences between chains, testing convergence for each parameter separately (while allowing for a Type-I error rate of 0.05 across all *p*-values). Model fit is evaluated by the posterior-predictive probability or *p*-value (*PPP*), which indicates the relative fit of model-generated data versus observed data, with values of 0.50 being optimal and values greater than 0.05 typically considered acceptable (Muthén and Asparouhov, 2012). Comparisons of models may be done using the deviance information criterion (DIC) as a relative index of model quality (balancing fit and parsimony), with smaller values indicating a better model. The DIC is useful because it is uniquely sensitive to the number of estimated parameters *pD*, which is a function of the number of unrestricted parameters and the amount of information provided by priors (see Asparouhov et al., 2015), and thus this value will typically not be an integer value as in the ML case where priors do not exist. Consistent with other approaches, we use the *SD* of posterior distributions to compute Bayesian analogs of two-tailed *p*-values (Zyphur and Oswald, 2015). For impulse responses, we use 95% credibility intervals with the highest posterior density, which are similar to bootstrap *CI*s (Rubin, 1981). For all parameters not explicitly mentioned, we use default uninformative/diffuse priors in Mplus (Asparouhov and Muthén, 2010), which is done for convenience and to keep the reader focused on the bespoke priors specification used here.

Model results are in Table 1 under the “Bayes 1” model, showing acceptable fit (*PPP* = 0.32). For concision, we report the averages and ranges of time-varying AR, MA, CL, and CLMA terms (readers can examine full results in our online materials). For example, AR effects for income have four terms associated with each occasion of measurement that is endogenous to all lagged effects, with $\beta_{x\,1,3}^{\left(x\right)}=0.97$, $\beta_{x\,1,4}^{\left(x\right)}=0.97$, $\beta_{x\,1,5}^{\left(x\right)}=0.94$, and $\beta_{x\,1,6}^{\left(x\right)}=1.0$, for which Table 1 shows the mean 0.97, the range [0.94, 1.0], and the average posterior *SD* = 0.03 (*p* < 0.001). As Table 1 shows, averaged terms are similar to their frequentist counterparts in many cases, such as the AR effect for the ML model $\beta_{x\,1}^{\left(x\right)}=0.96$ versus the Bayesian average 0.97. Furthermore, ranges are relatively small for Bayesian estimates, indicating little difference in most parameters over time under the combination of small-variance priors and observed data used here.

However, a noticeable change occurs in the level of uncertainty around parameters. For example, the AR effect for the ML model has an *SE* = 0.13, whereas the Bayes average has an *SD* = 0.03. This reduction in uncertainty is expected for two reasons. First, allowing parameters to vary over time can increase their fit to the data at each occasion, reducing uncertainty around the estimates as a function of better fit to the covariance for any two occasions. Second, as Table 1 shows, the total number of parameters estimated in the Bayes model is slightly smaller than the ML-based model (54 versus an estimated *pD* = 50.69 in the Bayes model), ostensibly because of the small-variance priors. In turn, although time-varying effects are allowed, the Bayes model appears to be slightly more parsimonious, implying less uncertainty for the entire model, which on average should result in smaller Bayesian posterior *SD*s than ML-based *SE*s.

An interesting consequence of this uncertainty reduction is that Granger-causality tests and impulse responses show different results for the income → SWB effect, supporting it much more strongly. In the ML-based model, the income → SWB effect is $\beta_{x\,1}^{\left(y\right)}+\delta_{x\,1}^{\left(y\right)}=0.14$, *SE* = 0.16, *p* = 0.40; whereas in the Bayes model it becomes $\beta_{x\,1}^{\left(y\right)}+\delta_{x\,1}^{\left(y\right)}=0.22$, *SD* = 0.12, *p* = 0.06. However, rather than relying on *p*-values, we test Granger-causality using the DIC. As shown in Table 2, the DIC for the full model is 829.23, and eliminating the income → SWB CL and CLMA terms increases this to 835.48, indicating reduced model quality and therefore supporting an income → SWB effect. Alternatively, removing the SWB → income CL and CLMA terms, the DIC falls to 820.83, indicating improved model quality and therefore failing to support an SWB → income effect. Finally, testing for income-SWB feedback by constraining all CL and CLMA terms also reduces model quality with a DIC of 833.35, providing support for feedback effects. Yet, this raises the question of whether the income → SWB effect is driving the larger DIC value when eliminating all CL and CLMA terms in order to test for feedback.

**FIGURE 2:**
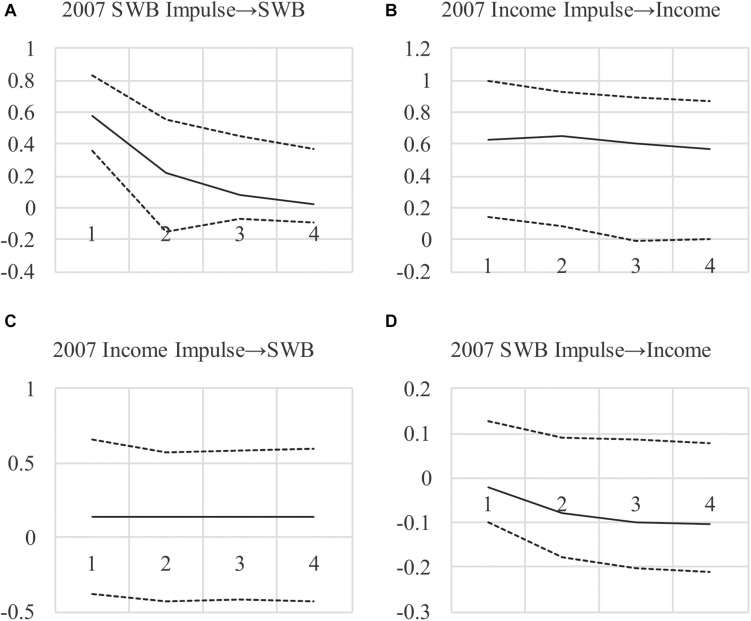
**(A–D)** Impulse Response Functions for AR(1)MA(2) Model With Bayesian Small-Priors. Impulse begin in 2007, showing the effect of a 1-unit impulse in 2007 over the next 4 years, with 95% credibility intervals (with the highest posterior density).

To investigate this and to show long-run effects, we examined impulse responses (see Figures 2A–D)^3^. The differences between the ML-based and Bayesian impulse responses are notable, with much less uncertainty around income’s persistence over time (the top-right figure). Also, impulse responses show a larger effect for income → SWB and much less uncertainty around the estimate, with 95% credibility intervals encompassing zero only at the margins (consistent with *p* = 0.06). Furthermore, the SWB → income effect is approximately zero across all periods. These findings lend more credibility to a positive long-run income → SWB effect when compared to the frequentist estimates in Figures 1A–D, and less credibility to a long-run SWB → income effect. The results also imply that the lower DIC value when testing feedback is due to the income → SWB effect rather than the opposite, arguing against income-SWB feedback.

In sum, the small-variance priors we use allow model specifications that are plausible yet under-identified with frequentist methods. By allowing effects to vary over time, we provide a better fit to the observed data and reduce the uncertainty around estimates, pointing to an effect of income on SWB that appears to be long-lasting. Indeed, when eliminating the SWB → income CL and CLMA effects, which is warranted based on the decrease in the DIC, we show an income → SWB effect combining CL and CLMA terms: $\beta_{x\,1}^{\left(y\right)}+\delta_{x\,1}^{\left(y\right)}=0.24$ with a posterior *SD* = 0.12, *p* = 0.04. Furthermore, this effect with a one-tailed test has a *p* = 0.02 and the 95% credibility interval in Figures 2A–D exclude zero. The implication is that a positive impulse to national income may have a positive immediate and long-run effect on SWB, neither of which was found in the ML-based analyses in Tables 1, 2, and Figures 1A–D, because of the restrictions on the effects that were required. For our “informal Bayesian,” this implies updated knowledge or belief about a causal income → SWB effect, which may be used to inform policy decisions.

### Reducing Lag Orders

To further tackle overparameterization and provide an additional tool for estimating models that may be under-identified, small-variance priors can be applied to high-order lags and unit effects. As with time-varying parameters, the issue is that estimating many lagged effects and time-varying unit effects can overfit observed data while also making models under-identified with frequentist estimators. This is important because, for prediction, “[e]vidence favors Bayesian estimation of an equation with high-order lags rather than restricted models arrived at by classical testing methods” (Allen and Fildes, 2001, p. 335; Stock and Watson, 2001).

To illustrate this approach while keeping our results both concise and comparable to those presented thus far, we specify the same models for both income and SWB (Eqs. 8 and 9), but set small-variance priors on the second-order MA lag for income. In Zyphur et al. (2020a) the authors appear compelled to choose a single model for income, comparing the results of AR(1)MA(1), AR(1)MA(2), AR(2)MA(1), and AR(2)MA(2) models for *x*_*i,t*_. Conveniently, a Bayes estimator changes the nature of this choice by allowing higher-order lags to have small-variance priors with means of zero, reflecting an expectation of no higher-order lagged effects while allowing them to emerge as a function of the data. The result is an ability to retain time-varying effects for AR, MA, CL, and CLMA terms while also allowing them to have many lags that minimally add to model complexity due to the use of small-variance priors. To show this, we set the following small-variance prior for the time-varying $\delta_{x\,2,t}^{\left(x\right)}$ term in Eq. 8: $\delta_{x\,2,t}^{\left(x\right)}∼N\,\left(0,0.01\right)$. This small-variance prior allows the second-order MA terms to vary over time while shrinking them toward zero and keeping the number of estimated parameters manageable.

The results of this model are shown in Table 1 under the “Bayes 2” model, with Granger causality tests in Table 2. As Table 1 shows, the fit of the model improves over the previous “Bayes 1” model that allowed the second-order MA term $\delta_{x\,2,t}^{\left(x\right)}$ to be unrestricted with a small-variance prior on differences with $\left(\delta_{x\,2,t}^{\left(x\right)}-\delta_{x\,2,t+1}^{\left(x\right)}\right)∼N\,\left(0,0.01\right)$. Specifically, the DIC falls from 829.23 to 827.65, indicating some improvement by shrinking second-order MA terms toward zero while still allowing them to be time-varying.

Interestingly, this second Bayes model also fits the data better than two others that may also seem warranted and of interest to researches exploring different MA structures for income. The first is a model wherein the same small-variance prior is applied but the second-order MA term is constrained to equality over time, with an effect $\delta_{x\,2}^{\left(x\right)}$ as in the original ML model in Table 1 and $\delta_{x\,2}^{\left(x\right)}∼\left(0,0.01\right)$. The DIC for this model increases to 831.46, arguing for the time-varying specification with the same null small-variance prior $\delta_{x\,2,t}^{\left(x\right)}∼N\,\left(0,0.01\right)$ in the Bayes 2 model in Table 1. The second model that seems plausible is one that takes the prior expectation of no effect as the actual model specification, fixing the second-order MA term to zero (i.e., fixing $\delta_{x\,2,t}^{\left(x\right)}\equiv 0$), resulting in a purely MA(1) specification for income. This model has a DIC that increases to 830.63, again favoring the MA(2) specification with the small-variance null prior on the second-order lagged MA effect $\delta_{x\,2,t}^{\left(x\right)}∼\left(0,0.01\right)$. In sum, the small-variance priors that allow time-varying effects outperform other plausible specifications in this case, and allow researchers to operationalize an expectation of no higher-order lagged effects while still allowing results to be pulled away from this prior expectation as a function of the data.

Given the improved fit of the second Bayes model in terms of the DIC, it is interesting to note that, again, Granger-causality tests under this model show an increase in the DIC when removing the income → SWB effect (see Table 2, Bayes 2 model), with the full model DIC = 827.65, but with all income → SWB CL and CLMA terms eliminated the DIC increases to 833.85. This is consistent with the overall income → SWB effect, which again is $\beta_{x\,1}^{\left(y\right)}+\delta_{x\,1}^{\left(y\right)}=0.22$, *SD* = 0.12, *p* = 0.06. Furthermore, as before, constraining the SWB → income CL and CLMA terms to zero improves model fit with DIC = 818.71, failing to support Granger-causality in this direction. Also, feedback effects appear to exist with DIC = 831.70 under a model with no CL and CLMA terms, but this appears to be entirely due to the income → SWB effect, which is supported by impulse responses, which we omit because they are very similar to Figures 2A–D.

In sum, there are at least three benefits of the informative, small-variance priors that we use here. First, they shrink estimates toward zero or toward each other for time-varying parameters, which in our model reduces uncertainty substantially and thereby supports an income → SWB effect that could not be supported with an ML estimator. This is useful because, second, the increase in model parsimony due to the model priors also increases generalizability, which means the income → SWB effect is also more trustworthy than under ML. Third, the priors we use allow estimation that would be impossible with a frequentist estimator, allowing higher-order AR, MA, CL, and CLMA effects while also using small-variance priors that reduce the need to choose amongst models that have different lag orders for these terms. These three benefits are in addition to those that apply to Bayesian estimation more generally, including not only its fit with an “informal Bayesian” using panel data models to make inferences under uncertainty, but also computational efficiencies of Bayesian estimation (see Chib, 2008; Muthén and Asparouhov, 2012; Zyphur and Oswald, 2015). For more details, the reader may consult additional work on Bayesian analysis for panel data models (e.g., Sims, 1980, 1986; Canova, 2007; Koop and Korobilis, 2010; Korobilis, 2013; Giannone et al., 2015; Schuurman et al., 2016).

## Discussion

In their recent series “From Data to Causes,” Zyphur et al. (2020a) described the GCLM parameters and their relationship to Granger causality and intervention planning via impulse responses, with all terms estimated via ML in an SEM framework. These authors also compared their approach to others, noting the benefits of dynamic models that make the future conditional on the past while controlling for unit effects, thus addressing issues with static approaches including latent curve models (i.e., latent growth or trajectory models; Zyphur et al., 2020b). However these authors did not acknowledge shortcomings of their frequentist estimation method and thus in the current article we extended the GCLM to the case of Bayesian estimation and inference, showing the usefulness of small-variance priors for both parameter estimates and parameter differences in models that would otherwise have high dimensions that produce generalizability and/or estimation problems. The result is that here we were able to estimate time-varying parameters while shrinking higher-order lagged effects and time-varying unit effects for income toward zero, reducing parameter uncertainty and allowing us to support an income → SWB effect that does not receive support under ML estimation.

With such Bayesian approaches to time-series and panel data modeling, researchers have a set of powerful tools for doing the practical work that defines the applied social sciences. This work has various characteristics that often center on theorizing and empirically studying causal effects, such as the income → SWB effect, which we support in the current study. For any applied science, the point of such a finding—and research more generally—is a practical affair, with researchers seeking to develop understandings of the world that can guide action, such as organizational or public policy interventions (Cartwright and Hardie, 2012). In turn, the point of these interventions is to create specific kinds of outcomes, such as improving SWB by helping poor nations to develop their economies in order to increase income. To these ends, a benefit of small-variance priors and methods of “shrinkage” more generally is to improve generalizability so that such inferences can have a greater chance of working in real-world situations.

However, there are various dangers associated with using models such as ours uncritically. One danger is the well-known problem of exactly how a researcher or policy maker should derive priors—what sources of information should be used for this purpose—and how the choice of different prior specifications may affect results. These topics have received substantial attention in Bayesian literature and we encourage the interested reader to engage with this work (again the interested reader may consult excellent work on these and other topics; e.g., Depaoli and van de Schoot, 2017; Smid et al., 2020). As we noted previously our use of the specific small-variance prior of ∼*N*(0,0.01) was used for example purposes and to fit with previous literature (see Muthén and Asparouhov, 2012; Zyphur and Oswald, 2015). Future work may investigate other potential types of small-variance priors to complement the existing and ever-growing body of work on the use of priors for Bayesian analysis of time-series and panel data models (e.g., Lüdtke et al., 2018).

## Data Availability Statement

The original contributions presented in the study are included in the article/Supplementary Material, further inquiries can be directed to the corresponding author.

## Author Contributions

MZ organized and led the research including analyses and initial manuscript drafting. EH, LT, MV, KP, ZZ, PA, DP, PK, and ED contributed substantial insights during the phases of model development and interpretation as well as generating conclusions in the discussion section. All authors contributed directly to manuscript writing and revisions during many collective rounds of editing and write-up.

## Conflict of Interest

The authors declare that the research was conducted in the absence of any commercial or financial relationships that could be construed as a potential conflict of interest.

## Footnotes

1 In order to identify a scale for η we fix one of each $\lambda_{t}^{\left(x\right)}$ and $\lambda_{t}^{\left(y\right)}$ terms to unity. In our previous papers and in our online Excel file that automates Mplus input, we did this for the final occasion λ_6_ = 1. Choosing this or any other occasion is an arbitrary decision, but in the current paper we set λ_1_ = 1 in order to facilitate some of the Bayesian prior specifications we describe.

2 As a form of sensitivity analysis, we also estimated a model with a small-variance prior for differences in income’s unit effects, setting $\left(\lambda_{t}^{\left(x\right)}-\lambda_{t+1}^{\left(x\right)}\right)∼\left(0,0.01\right)$ (for *t*> 1). We observed no notable differences in model results with this set of priors versus the prior $\left(\lambda_{t}^{\left(x\right)}\right)∼\left(0,0.01\right)$.

3 Time-varying AR, MA, CL, and CLMA effects imply a unique impulse response for each impulse over time. We calculate impulse responses based on the first available impulse given the model lag order MA(2). For discussion of impulse responses for time-varying parameters and Bayesian estimators, see Koop (1996).
